# The interplay between drug-use behaviors, settings, and access to care: a qualitative study exploring attitudes and experiences of crack cocaine users in Rio de Janeiro and São Paulo, Brazil

**DOI:** 10.1186/s12954-015-0059-9

**Published:** 2015-08-06

**Authors:** Noa Krawczyk, Carlos Linhares Veloso Filho, Francisco I. Bastos

**Affiliations:** FIOCRUZ—Oswaldo Cruz Foundation, Av. Brasil, 4365, Biblioteca de Manguinhos #229, Rio de Janeiro, 21045-900 Brazil; Psychiatry Institute, Federal University of Rio de Janeiro, Avenida Venceslau Brás, 71 - Fundos - Botafogo, Rio de Janeiro, RJ 22290-140 Brazil

**Keywords:** Crack cocaine, Stigma, Marginalization, Drug-use behaviors, Access to care, Health-service utilization, Risk environments, Qualitative analysis

## Abstract

**Background:**

Despite the growing attention surrounding crack cocaine use in Brazil, little is understood about crack users’ histories, use patterns and the interplay of drug-use behaviors, settings, and access/barriers to care. Qualitative studies seldom cross-compare findings regarding people who use crack from different settings. This study aims to explore the insights of regular crack users in two major Brazilian cities and to examine how social and contextual factors, including stigma and marginalization, influence initial use and a range of health and social issues.

**Methods:**

In-depth interviews and focus groups were conducted with 38 adult crack cocaine users recruited from impoverished neighborhoods in Rio de Janeiro and São Paulo. Interviews and focus groups were audio recorded and transcribed verbatim. Qualitative analysis was carried out, and content was organized and analyzed by recurrent themes relevant to study interests.

**Results:**

For study participants from both cities, frequent crack cocaine use plays a central role in daily life and leads to a range of physical, psychological, and social consequences. Common concerns among users include excessive crack use, engagement in risky habits, infrequent health service utilization, marginalization, and difficulty reducing use.

**Conclusions:**

Disadvantaged conditions in which many crack cocaine users grow up and live may perpetuate risk behaviors and stigma may further marginalize users from necessary health and recovery services. Reducing stigma and moralizing discourse related to drug use, especially among health professionals and law enforcement personnel, may help encourage users to seek necessary care. New harm-reduction-based care and treatment alternatives for marginalized drug users are being developed in parts of Brazil and elsewhere and should be adapted and expanded for other populations in need.

## Abstract (pt)

**Antecedentes:**

Apesar da crescente atenção em torno do uso de crack no Brasil, pouco se sabe sobre as histórias dos usuários, seus padrões de consumo, e a interação de comportamentos de consumo de droga, contextos e acesso/barreiras ao(s) cuidados de saúde. Estudos qualitativos raramente comparam os achados de pessoas que usam crack a partir de diferentes contextos. Este estudo tem como objetivo explorar os *insights* de usuários regulares de crack em duas grandes cidades brasileiras e examinar como fatores sociais e contextuais, incluindo o estigma e a marginalização, influenciam o uso inicial e uma variedade de problemas de saúde e sociais.

**Métodos:**

Entrevistas em profundidade e grupos focais foram realizados com 38 adultos usuários de crack recrutados em bairros pobres do Rio de Janeiro e São Paulo. As entrevistas e grupos focais foram gravadas em áudio e transcritas na íntegra. Procedeu-se à análise qualitativa e os conteúdos foram organizados e analisados por temas recorrentes relevantes para os interesses do estudo.

**Resultados:**

Para os participantes do estudo de ambas as cidades, o uso frequente de crack desempenha um papel central na vida diária e leva a uma série de consequências físicas, psicológicas e sociais. Os interesses comuns entre os usuários incluem o uso excessivo de crack, o engajamento em comportamentos de risco, a utilização de serviços de saúde pouco frequente, a marginalização e a dificuldade em reduzir o uso de drogas.

**Conclusões:**

As condições desfavoráveis em que muitos usuários de crack crescem e vivem podem perpetuar os comportamentos de risco, e o estigma marginaliza ainda mais os usuários dos serviços de saúde e de recuperação necessários. A redução do estigma e do discurso moralizante relacionado ao uso de drogas, especialmente entre os profissionais de saúde e policiais, pode ajudar a incentivar os usuários a procurar atendimento necessário. Novas alternativas de cuidado para usuários marginalizados, baseados em redução de danos estão sendo desenvolvidas em algumas localidades no Brasil e outros países, e deveriam ser adaptadas e expandidas para populações vulneráveis.

## Background

Crack cocaine (hereafter mentioned as “crack”) has received heightened attention by Brazilian citizens and governments due to its expanding markets and frequent use among visible homeless and impoverished populations [1]. Concern centers on the strong dependence it generates among users, often characterized by binge use and association with violence and risky sexual behaviors [2–6], as well as high rates of infectious diseases and other health conditions present among this population [7]. While drug use and consequent habituation, dependency, and related behaviors have been associated with a multitude of factors [8], use is commonly higher in disadvantaged environments with high accessibility to drugs and low economic and educational opportunities [9, 10]. In Brazil, most crack users come from underprivileged socioeconomic backgrounds and continue to live in poverty and unstable conditions: the recent epidemiological National Survey of Crack Users in Brazil, which conducted over 7300 interviews throughout the country, found that over 80 % (rounded to the nearest integer) of crack users had not reached a high school level education and that 80 % are identified as non-white. Only 36 % of users lived in their own houses/apartments, 18 % lived with friends/acquaintances, 4 % lived in provisional hostels/rented rooms paid for daily/shelters, and 39 % lived on the streets. Moreover, the vast majority of interviewees (97 %) had no formal employment, with a substantial fraction of people holding temporary jobs (68 %) and a noteworthy percent of people who pan handled (13 %), and people who admitted to having been involved in illicit activities (e.g., drug-dealing, muggings, etc.; 8 %) [11].

Economic and social factors not only affect initial drug use but also influence the severity of behaviors associated with use and users’ access to care [12, 13]. While Brazil’s federal health-care network, the Unified Health System (SUS), is incorporated into the Brazilian constitution and guarantees free health care to all citizens [14], crack users rarely utilize health services [5, 14–18]. The above national survey documented that only 27 % of participants had utilized some type of health service in the month previous to the interview [19]. Low service utilization among drug users is common and has been attributed to poverty, unstable housing, frequent mobility, and other elements that often characterize marginalized groups [12, 20, 21]. Barriers expressed by Brazilian crack users include limited service resources, lack of needs-specific professional skills among health providers, bureaucratic obstacles, and stigma [16]. Stigma associated with alcohol, drug use, and unstable housing conditions has been shown to prevent access and adherence to health and treatment services by discouraging users from seeking care [22–26].

Crack use has historically carried particularly elevated stigma: in the United States, crack use among poor and inner city people of color in the 1980s resulted in widespread concern regarding drug use and public safety. Such panic was sustained by harsh drug laws and unfounded claims that asserted crack was the determinant of multiple social and medical ills including the so-called crack babies symptoms, which were later recognized to have stemmed from poverty, under-nutrition, and syphilis, among others [27, 28]. The Brazilian media’s recent claims of a crack epidemic have led to similar accusations that criminalize and marginalize users [29]. Moreover, poor drug users are common targets of police violence and discrimination. Brazil has a long-existing tradition of highly militarized state police forces, aggravated by a 21-year-long military dictatorship (1964–1985) in which local police forces were directly trained by military forces and adopted their doctrine of stringent and often violent national security [30]. Abusive acts perpetrated by members of the police are frequent, even among new police cohorts [31]. Consequently, although the act of sleeping in public spaces and the possession of small amounts of drugs for personal use are not, according to federal law, considered crimes in Brazil [32], drug sales are still highly criminalized. Books published by criminologist Luciana Boiteux and other Brazilian scholars have documented that despite the formal distinction between personal use and trafficking, current legislation remains confusing. In the absence of clear psychological and/or behavioral parameters or objective limits defining legal possession for personal use vs. trafficking, laws are enforced at the individual policemen’s discretion, with unfortunate consequences such as mass incarceration and denial of treatment for drug users in need [33].

The stigma related to drug use and the conflicting policies taken up by national and local governments throughout Brazil have made it difficult to reach users and provide effective services that cater to their needs. Through SUS, the federal government has vowed to promote harm reduction as a method of dealing with drug and alcohol use throughout Brazil and has expanded out-patient psychosocial clinics for alcohol and drug use treatment as well as mobile clinics that do outreach and harm reduction efforts with homeless or hard-to-reach communities [34]. Still, these services are limited in number and in scope and are contradicted by efforts of local state governments that have periodically carried out mandatory removal of users to shelters and internment in private rehabilitation centers [35]. Such contradictions and the alienation of Brazilian crack users and has made it difficult to learn about their individual histories, use patterns, and the role that stigma and social factors play in drug-use behaviors and access to care. Such knowledge is crucial to planning appropriate public health strategies that incorporate users’ social and environmental contexts.

Rio de Janeiro (RJ) and São Paulo (SP), Brazil’s two greatest metropolises, contain large drug scenes. However, the scenes’ spatial-structural makeups differ greatly between cities [36, 37] as do their histories and public policies towards drug users and their available public health and social services networks [38–41]. While some qualitative studies have been conducted with crack users in SP [3, 42, 43], few have explored users’ experiences in RJ. This qualitative study used in-depth semi-structured interviews to explore the insights of regular crack users in both cities and to understand how social and environmental factors, including stigma and marginalization, influence initial use as well as a range of temporary and permanent health and social issues.

## Methods

In-depth qualitative interviews and focus groups were conducted with adult crack users in RJ and SP between April and June of 2012. They were carried out simultaneously and as a complement to the aforementioned epidemiological National Survey of Crack Users in Brazil that conducted quantitative interviews with over 7300 crack users recruited by word-of-mouth in open drug-use scenes across the country [44]. The participants of the qualitative interviews and focus groups did not necessarily take part in the quantitative component of the National Survey. However, they were recruited from the same scenes and thus were part of the same population subgroup. Recruitment took place with the support of local community leaders (members of community organizations and/or health and social work professionals that work in the area) that helped secure adequate spaces for carrying out interviews and focus groups.

This qualitative study accessed a convenience sample of individuals who regularly use crack and a selection of participants was done intentionally (non-randomly), with basic criteria to attempt to choose people of different age groups and genders but without aiming to statistically represent the population of crack users at a larger scope. Participation eligibility criteria included being over 18, using crack regularly as defined by the Pan American Health Organization’s CODAR criteria (for at least 25 days in the last 6 months) [45], and consenting in writing to participate in the study. Those who were acutely intoxicated or had delusions and/or other acute mental health conditions at time of interview were excluded from participation. Participants who completed the interview were compensated with 20 Brazilian Reals (~US$10, at time of study). In addition, in every drug scene in which research was conducted, recruiters offered voluntary HIV and hepatitis C testing services in local public primary care units.

The team of interviewers in each city was comprised of two researchers, usually psychologists or social workers by profession, who had been previously trained to conduct theory-driven qualitative interviews and focus groups by the research coordinators at the Oswaldo Cruz Foundation in Rio de Janeiro, two of which are authors of this paper. Individual interviews and focus groups were designed to be short and last an average of 30 min in order to encourage subject participation and deter participants from leaving the conversation in the middle. Individual interviews followed a semi-structured questionnaire and focus groups followed a list of discussion topics, both which explored crack and other drug consumption patterns, including childhood and family history of drug use, motivation to initiate drug use, social norms among crack users, violence (as perpetrator and victim), perception of stigma and discrimination, availability and utilization of health and social services, perceptions about current health, and future aspirations. Participants were encouraged to comment freely about matters of interest. Interviews and focus groups were audio-recorded and later transcribed by authors verbatim.

The authors analyzed narratives using principles of content analysis [46] with the help of the Atlas.ti® software. Both audio and transcribed versions were used in the analysis in order to incorporate the tone of participants as they related their experiences. Codes were originally created using interview questions/focus group topics as a guide and were modified as information emerged from the narratives. Due to the nature of the focus groups in which participants spoke freely, comments could not always be linked to a specific participant and thus sentiments expressed were not quantified. Instead, extracted content was organized by recurrent themes relevant to study interests. General notions were paraphrased and selected quotes were translated by authors (one native English and one native Portuguese speaker) and included to illustrate results. The study protocol was approved by the Ethical Review Committee of FIOCRUZ.

## Results

Thirty-eight respondents participated in this study whose narratives were included in the analysis. Participation took place through four semi-structured in-depth individual interviews for each city (two males and two females) and three focus groups for each city (one all-male, one all-female, and one mixed-gender, each containing three to six participants). Most participants indicated being poly-drug users and length of crack-cocaine use specifically ranged from 1 to 14 years. Table 1 summarizes selected characteristics of the sample. Interview content was organized according to the following thematic categories that emerged in the analysis and selected quotes are included in each section below.

**Table 1:** Study participant characteristics

	São Paulo		Rio de Janeiro	
	Number of participants	Ages	Number of participants	Ages
Individual interview female	2	30; 38	2	27; 36
Individual interview male	2	22; 23	2	24; 30
Focus group female	6	30; 31; 32; 33; 38; 39	6	21; 27; 28; 31; 40; 48
Focus group male	5	20; 23; 27; 40; 41	5	27; 30; 35; 36; 36
Focus group mixed	3	29; 33; 38	5	23; 26; 27; 28; 32

### Crack use habits

Respondents described recurrent crack use, obsessive thoughts about use and craving and withdrawal feelings when not using. For example, participants talked about feeling abstinent from crack, described that when they didn’t smoke they felt anxious and agonized about when they will have more crack and felt strong desires to smoke again. One participant from RJ said the second week without crack she started to dream and hallucinate about using crack and started to smell the scent of it, which caused her to desperately want to smoke again. Respondents described losing former interests and that crack negatively interfered in their personal and professional lives. Many indicated having continued use despite various attempts to stop or reduce intake. Marcos (names are fictitious here and in all subsequent quotations) (30, RJ) described how his life became focused on crack:

Today? My job is to smoke crack. To work? Nope, not to work, to get money for crack.

Respondents explained that to acquire crack, some users sell their personal possessions, manipulate, steal, or engage in prostitution. One woman in a female focus group in RJ explained:

To buy crack, you—well, us who are users—we have several options: either we rob, or we prostitute ourselves, you understand?

Renan (22, SP) described that users can end up stealing from their very families, so he chose to leave home to avoid this:

I left home so that I wouldn’t make her suffer—so that I wouldn’t steal from her, from my siblings. Because I was already feeling that craving and need to want to use and I didn’t have the money and stuff, so then I thought and was like: ‘I’m going to leave my mom’s house or else I will end up screwing up, messing up with her, and I can’t do that.’ So I left.

In a SP focus group, one respondent described how some users become so involved in using crack they neglect to care for their basic necessities:

It’s like this: people who haven’t adapted to or know how to use crack yet, they come here to use and then they’re done. They forget to eat, forget to shower, forget everything and just end up smoking, smoking, smoking!

### Facilitators of crack use

Respondents spoke of personal and environmental factors that facilitated access to drugs and eventually led to crack use. Some mentioned growing up with family members who used drugs. Others described using drugs as a form of dealing with emotional problems, family issues, or other traumatic experiences. One woman from SP described how crack use helped alleviate emotional pain:

When I used for the first time, all the pain I was feeling at that time…because of the end of my engagement and all the humiliation… I felt alleviated from this pain. Because crack, honestly, it takes away some of your emotions. It takes away your emotions—just takes them away!

Another woman from RJ described beginning crack use as a way of dealing with traumatic and violent events:

They stole my son, killed my brother, I lost my father, they killed my sister… My use was in revolt. Mostly revolting because they stole my son from my arms in the maternity ward, you know?! My brother—they lit by brother on fire alive! My brother was innocent, you know?! My father died from an overdose in my arms…on the way to the hospital. They raped my sister in the favela^1^. My use was in revolt. Definitely a lot of revolting.

Although many connected drug use to problematic families and life events, others asserted that they had good upbringings and that their use was unrelated. Some attributed crack use to growing up in environments where drugs were easily available and used in social circles. Several mentioned being exposed to crack in poorer communities where drug presence, crime, and violence are common. One respondent from RJ described access to drugs in his community:

I lived in the slum—where my entire family still lives today—there in Cidade Alta^2^ […] So contact with drugs, even more so for one that lives in a slum […] is constant.

Carla (RJ, 26) described how the lack of opportunities available for people from poorer communities facilitated involvement in crime and drug trafficking:

So what do you expect? That no one rob? That no one sell drugs? That no one watch out for themselves? That no one panhandle in front of the Garotinho^3^? Why not? If they at the top don’t even give us the opportunity to work!

### Crack user stigma and marginalization

Almost all respondents felt stigmatized for using crack and stated that society described them using derogatory terms like “Cracudo^4^,” “Craqueiro,” or “Noia.^5^” As Renan (22, SP) described:

Those who don’t use themselves think people who use are all “Noias”. Noia means a low thief, one who keeps begging for things on the street—that is a Noia. A Noia does everything wrong: steals from his friends, steals from his family. And with crack, if you don’t have it under control, you do this, my friend! You steal from your mother, your father, you steal from anyone and everyone around you. You steal so you can use the drug. It’s a craving, a craving and the need to use more, and more, and more!

Another respondent described that discrimination stems largely from their street presence and destitute appearance of users:

Society sees us as Noias, right? Not just because of the drug. Since it’s an addictive drug, the person who’s a Noia sells their cap, sells their shirt, sells their flip flips, sells their shoes. And when one finally goes to look at themselves—they look absolutely horrible: barefoot, torn shorts… Some Noias—I won’t say that I’m a Noia, I am a user! … And because people are badly dressed—like horribly—without having showered…society sees them as garbage. So there is a lot of prejudice.

Respondents described that often users are wrongly labeled as thieves, sick, or undeserving, and some, like Adriana (30, SP), had experienced violent attacks by regular citizens or the police:

We go anywhere and people want to beat on us […] Me—myself—the first time I went through this, I went to ask for R$0.50 from a guy on the street, and the guy said to me: “Get a job, you bum!” Started cursing at me, he came at me with everything! And so I responded…I called the police to come and they came and you know what they did? They grabbed me by force, grabbed my arm, and they beat me all up. The police beat me up. […] I’m just another crack user, I have no credit, no value, I don’t have a say, you know? Even though I know I have it within me, to society, today I don’t have one. Today, I don’t have a life.

### Discrimination in health services

In RJ, most respondents expressed frustration and cynicism regarding care at public health centers. Most had experienced discrimination from staff, including being ignored or treated rudely or with disgust. As a woman in a RJ focus group described:

Everywhere, UPA^6^ (Urgency Health Centers), places like that—are horrible. When the sick person gets there, if he’s sick enough to die, he will die waiting. Also, doctors don’t even look you in the face, you know? They also treat the person with disgust. You know why? Because it’s like this: Just because we use this drug—this darn drug—they are prejudice against us. And because of this, they are too grossed out to even touch us. I always tell them “we are humans just like all of you!” To me, all these [services] are one big piece of crap.

In contrast, in SP, most respondents expressed positive experiences with public health services, stating that staff treated them with attention and care. Many were more familiar with services offered at centers like the public psychosocial clinics (CAPS^7^) and sexual health clinics and said they had frequent contact with street-outreach^8^ health agents. Still, others noted problems including limited resources, complex bureaucracy, and bad experiences with staff. While most respondents knew how to access health services, many indicated that they only sought care in worst-case scenarios. As Bruno (22, SP) explained:

Here there are always several health agents passing by, walking around, and [users] just don’t pay attention. They don’t have a minimal interest in them. The crack user only seeks out a doctor when he is really sick. But if the health agent passes by for any other reason and says—“oh, sir, can I speak to you?” It’s “No, no! Wait, hang on—I am smoking my crack over here” and he doesn’t even pay any attention.

### Self-blame and exclusion by crack users

A majority of respondents expressed strong regret at how crack use has affected their lives. Some, like Marcos (30, RJ) expressed guilt for having abandoned or hurt their families and too ashamed or dependent on drugs to return:

I used to go down to the park with my daughter, to enjoy some free time, too. And nowadays, what am I now, you know?! Damn, there are days when I wake up crying, alone, and missing my daughter. Just saying her name my eyes begin to water and I miss her so much. Damn.

Several respondents referred to their state as vile, using demonizing and criminalizing language to describe themselves. As one respondent described:

I work for crack. I bribe for crack. I manipulate for crack. The worst manipulator out there is the Cracudo himself, the Craqueiro.

One woman in a RJ focus group described crack use by repeating a statement she heard on a popular radio show:

Crack makes you kill your mom and dad, your son, mom, grandmother, grandfather. Yes, crack. Crack is causing a lot of things, you know? Now, what are we hearing about powder cocaine? Is someone raping for powder cocaine? But for crack…it’s what the guys even say on Tupi,^9^ so…

Respondents also described discrimination among users based on factors such as class, health status, appearance, where they live, and how they make a living. Some mentioned mistrusting other users as well as crime and manipulation between users. Simultaneously, many acknowledged the social nature of crack use: most said they used and shared crack in groups and with friends. One man from RJ described how crack unites people of different backgrounds:

There is no social class, no middle class, no high class, no low class. It’s among everyone, you know? Everyone. I’ve already smoked with a lawyer, I’ve smoked with a male judge, a female judge. I even smoked crack—I swear—with a professional soccer player.

### Desires for treatment and recovery

Most respondents expressed a desire to improve their situation or recuperate their previous lifestyle (i.e., when their daily life was not centered on seeking, getting, and using crack). Many respondents stated they wanted to stop or reduce crack use and indicated plans to do so in the future but stated that they currently were not ready to change their lifestyle. Almost all respondents already participated in some kind of treatment, including faith-based treatments,^10^ in-patient research-related programs,^11^ self-help groups like Narcotics Anonymous, and spiritual/herbal therapy using *ayahuasca*^12^*.* Some indicated good experiences with treatment and having stopped use for as long as 7 years; others stated they failed several treatments by relapsing back into use. Respondents provided several suggestions for what they believed a successful treatment or recovery service would comprise, ranging from individualized therapy, to isolation from drugs to job-training/placement services. Some respondents stated disbelief in any treatment. Despite varying opinions, most mentioned that personal desire and strong support systems are critical in treatment. Bruno (22, SP) described:

I am going to quit because there are people who love me, because the worst thing for a crack user is when you are rejected because you don’t need to be rejected, you need someone to love you, someone to incentivize you and tell you: “Man, don’t do this.” Crack is like that, if we wouldn’t have someone supporting us, that we see that loves us, that incentivizes us to get out of this… The more we are rejected, the more we want to use.

## Discussion

This study sought to explore the experiences and insights of crack users in two major Brazilian cities. Many participants stated feeling dependent on crack, experiencing frequent cravings and desires for use. Strong cravings for use, likely generated by crack’s quick and immediate effect, short duration, and tendency to be used in large amounts in short periods of time (binging) [47], have been widely reported by crack users [3, 42, 48]. While participants described different crack use habits and varying opinions on topics discussed, common concerns included excessive crack use, engagement in risky habits, infrequent health service utilization, marginalization, and difficulty reducing use. Similar experiences were found in qualitative studies conducted with crack users from other Brazilian regions [15, 43, 49, 50]. Most participants recognized the negative impact of use but indicated that despite wanting to, they had not been able to stop or reduce use. Throughout narratives, it became apparent that many of the participants’ personal histories, social circumstances, and ongoing marginality play a major role in the harms related to their drug use and in preventing their access to effective services. How addictive behaviors are sociologically contingent and how outcomes of drug use vary according to one’s position in a social structure has been recently explored and consolidated by Granfield and Reinarman [51].

Respondents’ personal histories revealed that many had been exposed to circumstances that, when combined with easy availability of drugs, were likely to elevate risk of substance use and abuse. These include exposure to violence, family history of alcoholism/drug-use, family conflict, social influences, and alienation and rebelliousness [9]. Violence and conflict are common in Brazilian impoverished communities and slums, mostly known as *favelas*, in which many of the study’s participants described growing up. Such communities have endemically suffered from structural marginalization that has limited economic opportunities and opened the door for drug trafficking, corruption, and violence from both trafficking groups and police [52]. Women often bear a disproportionate burden of the violence in such communities and are more likely to experience both physical and sexual intimate partner violence in communities where male-to-male violence is high [53]. Some female participants in Rio de Janeiro described using drugs to alleviate pain from such emotional traumas, even though it led them into a greater state of vulnerability. Indeed, female crack users, commonly involved in sex work, are likely to experience violence by clients, partners, and law enforcement and are at higher risk for HIV infection and other health burdens in Brazil and elsewhere [15, 54, 55].

Respondents expressed feeling stigmatized by citizens, authorities, and health professionals, often internalizing negative notions about crack use and expressing shame at their own wretched state. Respondents often referred to themselves as *Cracudos* and *Noias* and demonized themselves and other users. Participants often generalized that crack users have no control over their actions even when relating cases in which they clearly do, such as Renan (22, SP) that had left his family’s home in order to avoid hurting them. It is possible that even the self-neglect and failure to take care of one’s own hygiene described by participants may partially occur as a form of internalizing discrimination and humiliation by users. Several described avoiding contact with mainstream social circles and feeling less discrimination in the *cracolândia,*^13^ where open crack use is accepted and they can socialize with others. Such exclusion has been described by other drug-using groups that often choose to isolate themselves in order to flee persecution and discrimination [25].

Another form of social isolation discussed by participants was low health and social service utilization. Despite claiming poor physical and mental health, most participants that are aware of public services chose not to utilize them. Respondents attributed this phenomenon as stemming from both crack use’s role in diminishing their desire to care for their bodies and from previous bad experiences with health services. Negative attitudes towards substance users in health facilities have been described as contributing to poor health, diminishing empowerment and worsening treatment outcomes [56]. In his ethnography of injection drug users in San Francisco, Bourgois explains that even the most devoted medical practitioners became frustrated with drug users who complicate their already-severe medical problems and abuse efforts of those who try to help [25]. In Brazil, such frustrations may be exacerbated by the conditions of often over-worked and under-trained staff at public health facilities. Malta et al. found that public health professionals in RJ tend to lack proper training to work with drug users and have low prioritization of vulnerable populations [57]. Horto et al. revealed that health professionals who work with crack users in psychosocial care centers (CAPS) in Southern Brazil felt overworked and unsatisfied with their jobs, affecting the service quality they provided [58]. Interestingly, while respondents from both cities stated that they generally reach out for medical help solely in emergencies, RJ respondents more often emphasized being disrespected and humiliated by health services staff. In contrast, respondents in SP described frequent contact with health professionals and recognized the high availability and quality of public programs and services, demonstrating that outreach efforts may have been effective in making services that cater to users’ needs widely known.

The difference in service experiences described by participants from RJ and SP may be due to variations in policies and approaches to drug use taken by the two municipalities in recent years. Although Brazil’s Federal Ministry of Health governs health-care law, individual state and municipal governments have substantial jurisdiction in implementing their own policies and allocating funds towards programs of interest in regards to drug use. Thus, at different times, governments with differing political views have more strongly supported certain programs over others. In recent years, the municipal government of São Paulo has supported a series of progressive programs that have increased the presence of harm-reduction, social services, and mobile health clinics in areas of heavy crack, including the “De Braços Abertos” program that provides housing, job placement, and social and health services for users [59]. These services have largely been concentrated near one large crack use scene in São Paulo city, from which participants in this study were recruited, and may explain why so many participants in São Paulo have had better and more frequent experiences with outreach health-care workers. In Rio de Janeiro, some mobile clinics do similar harm-reduction and outreach work, but crack-use scenes are smaller and more dispersed throughout Rio de Janeiro city, which may explain why less participants in this study had contact with such services. Moreover, leading up to the 2014 World Cup, the government of Rio de Janeiro supported a series of occupation of poor neighborhoods by the police [31] and the compulsory removal and detention of homeless drug users, including minors, to shelters and private in-patient treatment centers [35] (this was later suspended due to harsh national and international critique of human rights violations [60]). In addition to further stigmatizing and isolating drug users, these actions diminished trust between municipal health-care workers and crack users and interfered with genuine efforts to connect users with urgent health and harm reduction services.

Most respondents expressed a desire to stop, reduce, or better control their crack use, but had mixed opinions regarding what kind of services could help their recovery. Some believed that only spiritual and religious faith can help improve their condition, highlighting the importance of considering religious beliefs when designing services for users. Feelings of failure expressed by users that had relapsed from abstinence-based treatments highlight the need to build programs that focus on improving the overall wellness of crack users and accepting relapse as part of the recovery process. Studies in SP reveal that some crack users have found ways to independently control use and engage in activities that alleviate the desire to use crack [3, 42]. We can learn from such experiences to design realistic techniques and recovery plans. Services that offer multiple treatment modalities with a combination of abstinence and harm reduction strategies have already been associated with positive outcomes among some substance-using groups [61]. In addition, efforts should be invested in learning from new treatment and care alternatives that work to cater to the needs and desires of users, including the previously described De Bracos Abertos program in São Paulo [59] that provides a range of health and social services for marginalized drug users in line with other successful housing first intervention-based programs for homeless, ethno-racial, and mentally ill groups [62, 63]. Other noteworthy example of progressive efforts in Brazil to provide health and harm reduction services to homeless and marginalized users is the work of mobile outreach street teams, although their influence is still restricted by limited training and resources [64].

This qualitative study is by its nature limited, and we cannot attest nor wish to suggest that attitudes and experiences are representative of crack users in RJ and SP or greater Brazil. Moreover, all information was self-reported and social desirability dynamics may have influenced responses and attitudes. Study designers attempted to avoid bias by using a combination of focus groups and individual interviews and by ensuring respondent anonymity.

## Conclusions

This study revealed important insights about experiences and perceptions of crack users that cannot be gathered from large-scale and purely epidemiological data. For study participants, frequent crack use plays a central role in daily life and leads to physical, psychological, and social consequences. The disadvantaged conditions in which many respondents grew up and continue to live significantly perpetuate risk behaviors and discrimination against crack users further marginalizing them from necessary health and recovery services. These narratives highlight the importance of addressing environmental factors related to substance use and creating alternative opportunities for people in high-risk communities. Efforts must be aimed at reducing stigma and moralizing discourse related to drug use, especially among health professionals and law enforcement, as well as improving conditions in public health facilities catering to vulnerable populations. New and progressive harm-reduction-based care and treatment alternatives for marginalized drug users are being developed in certain parts of Brazil and elsewhere and should be adapted and expanded for other populations in need. Further in-depth qualitative studies of this nature can help us better understand how individual experiences and social contexts relate to the manifestations of crack use and dependence and should be utilized to inform valuable health and social service strategies to alleviate the harms of drug use and help reintegrate marginalized users throughout Brazil and elsewhere.

## Footnotes

1 *Favelas* are the name for slums in Brazil, historically associated with poverty, violence, drug trafficking, and bad relations with the police.

2 Cidade Alta is a *favela* in the North Side of Rio de Janeiro with heavy presence of drug trafficking groups.

3 *Garotinho* is a nickname used for subsidized soup kitchens offering R$1.00 (~US$0.50) meals that were implemented by former Governor Anthony Garotinho and often utilized by poor and/or homeless citizens.

4 *Cracudo* and *Craqueiro* are derogatory terms used mostly to describe crack users who frequent the street. These expressions are similar to the English use of expressions that embody a given behavior (e.g., “pothead” or “crackhead”) as an intrinsic characteristic of someone, as opposed to (in principle, neutral) denominations as “users of crack cocaine.”

5 The term *Noia* is derived from “paranoia” and used to derogatorily describe drug users and the paranoid/delusional behavior associated with their behavior.

6 *UPA* or *Unidades de Pronto Atendimento* are 24-h free urgent care units that make up part of the Brazilian Unified Health System (UHS) [65].

7 *CAPS* are psychosocial health clinics of the UHS that specialize in mental health care and/or drug abuse services [66].

8 Street outreach clinics known as *Consultório na Rua* are specialized and often mobile primary care units of the UHS where staff go into drug-use scenes to offer basic care and harm reduction services to homeless/street-frequenting populations who have little contact with the formal health system. Such units were expanded as part of a federal program to deal with crack use in Brazil “*crack é possível vencer*” [67].

9 *Super Rádio Tupi* is a popular radio station frequently streamed throughout Rio de Janeiro.

10 Religious institutions that offer abstinence-based residential treatment and recovery programs are common throughout Brazil and are known as “Therapeutic Communities.” These institutions are privately funded and/or partially subsidized by the State.

11 Throughout Brazil, many clinics that offer treatment and services for drug users are affiliated with university research institutions and integrate findings from the clinic’s practice into research studies. For example, the Federal University of São Paulo (UNIFESP)-affiliated center for alcohol and drug-research, known as UNIAD [68].

12 Ayahuasca is a drink made from Amazonian plants containing the psychoactive compound dimethyltryptamine and used by certain religious denominations in Brazil to treat symptoms of drug dependence [69].

13 *Cracolândia* is a term coined by the Brazilian media to describe large and open-air congregations of crack users. Such large drug-use scenes are most common in Southeastern cities like São Paulo and Rio de Janeiro.
